# Nsp1 protein of SARS-CoV-2 disrupts the mRNA export machinery to inhibit host gene expression

**DOI:** 10.1126/sciadv.abe7386

**Published:** 2021-02-05

**Authors:** Ke Zhang, Lisa Miorin, Tadashi Makio, Ishmael Dehghan, Shengyan Gao, Yihu Xie, Hualin Zhong, Matthew Esparza, Thomas Kehrer, Anil Kumar, Tom C. Hobman, Christopher Ptak, Boning Gao, John D. Minna, Zhijian Chen, Adolfo García-Sastre, Yi Ren, Richard W. Wozniak, Beatriz M.A. Fontoura

**Affiliations:** 1 Department of Cell Biology, University of Texas Southwestern Medical Center, Dallas, TX 75390, USA.; 2 Department of Microbiology, Icahn School of Medicine at Mount Sinai, New York, NY 10029, USA.; 3 Global Health and Emerging Pathogens Institute, Icahn School of Medicine at Mount Sinai, New York, NY 10029, USA.; 4 Department of Cell Biology, University of Alberta, Edmonton, AB T6G 2H7, Canada.; 5 Department of Molecular Biology, University of Texas Southwestern Medical Center, Dallas, TX 75390, USA.; 6 Howard Hughes Medical Institute, University of Texas Southwestern Medical Center, Dallas, TX 75390, USA.; 7 Department of Biochemistry, Vanderbilt University School of Medicine, Nashville, TN 37235, USA.; 8 Department of Biological Sciences, Hunter College, New York, NY 10065, USA.; 9 Graduate School of Biomedical Sciences, Icahn School of Medicine at Mount Sinai, New York, NY 10029, USA.; 10 Nancy B. and Jake L. Hamon Center for Therapeutic Oncology Research, University of Texas Southwestern Medical Center, Dallas, TX 75390, USA.; 11 Departments of Internal Medicine and Pharmacology, University of Texas Southwestern Medical Center, Dallas, TX 75390, USA.; 12 Department of Medicine, Division of Infectious Diseases, Icahn School of Medicine at Mount Sinai, New York, NY 10029, USA.; 13 The Tisch Cancer Institute, Icahn School of Medicine at Mount Sinai, New York, NY 10029, USA.

## Abstract

The ongoing unprecedented severe acute respiratory syndrome caused by the SARS-CoV-2 outbreak worldwide has highlighted the need for understanding viral-host interactions involved in mechanisms of virulence. Here, we show that the virulence factor Nsp1 protein of SARS-CoV-2 interacts with the host messenger RNA (mRNA) export receptor heterodimer NXF1-NXT1, which is responsible for nuclear export of cellular mRNAs. Nsp1 prevents proper binding of NXF1 to mRNA export adaptors and NXF1 docking at the nuclear pore complex. As a result, a significant number of cellular mRNAs are retained in the nucleus during infection. Increased levels of NXF1 rescues the Nsp1-mediated mRNA export block and inhibits SARS-CoV-2 infection. Thus, antagonizing the Nsp1 inhibitory function on mRNA export may represent a strategy to restoring proper antiviral host gene expression in infected cells.

## Teaser

SARS-CoV-2 Nsp1 protein binds the mRNA export receptor NXF1-NXT1 to inhibit nuclear export of mRNAs and promote infection.

## INTRODUCTION

**Fig. 1:**
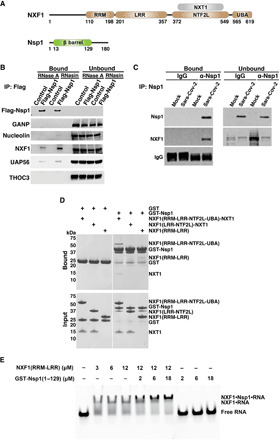
Nsp1 protein of SARS-CoV-2 interacts with the mRNA nuclear export receptor NXF1. (**A**) Schematic representation of NXF1-NXT1 and Nsp1. NXF1 contains an N-terminal RNA recognition motif (RRM), a leucine-rich repeat domain (LRR), a nuclear transport factor 2-like domain (NTF2L), and a ubiquitin-associated domain (UBA). RRM and LRR domains form an RNA binding module. NXT1 forms a heterodimer with NXF1 by binding to the NTF2L domain of NXF1. Nsp1 contains a β barrel domain. (**B**) 293T cells were transfected with 3xFlag-Nsp1 for 24 hours and then subjected to fractionation into nuclear and cytoplasmic fractions. See fig. S1 for fractionation controls. Nuclear lysates were subjected to immunoprecipitation (IP), followed by Western blot analysis to detect the indicated proteins. *n =* 3. (**C**) 293T cells expressing the SARS-CoV-2 receptor ACE2 protein were infected with SARS-CoV-2 at an MOI of 1 for 24 hours. Nsp1 was specifically immunoprecipitated from the cell lysates, and NXF1 interaction was detected by Western blot analysis. *n =* 3. (**D**) In vitro GST pull-down assays using the depicted purified recombinant proteins show that Nsp1 directly binds to NXF1. *n =* 3. (**E**) NXF1 associates with RNA in the presence of Nsp1. An electrophoretic mobility assay was carried out with a fluorescently labeled poly(U) 15-mer RNA and purified recombinant NXF1 (RRM-LRR) and/or Nsp1 (1-129) as indicated. *n =* 3.

Viruses inhibit host gene expression pathways to favor their own replication. Usually, these processes involve the action of multifunctional virulence factors that inhibit host gene expression at multiple levels to down-regulate immunity such as preventing interferon (IFN) response. A well-known example is NS1 protein, a virulence factor of influenza viruses, which inhibits cellular antiviral gene expression by targeting several cellular processes (*1*). A key pathway targeted by NS1 is the cellular messenger RNA (mRNA) nuclear export machinery. We showed that NS1 directly interacts with the mRNA export receptor heterodimer NXF1-NXT1 (*2*–*4*). During the early stages of mRNA export, cellular mRNAs are bound by export factors such as the THO complex and Aly/REF (THOC4), which initiate the mRNA nuclear export process (*5*, *6*). These export factors recruit NXF1-NXT1, which then facilitates mRNA translocation through the nuclear pore complex (NPC) to the cytoplasm for translation (Fig. 1) (*5*, *6*). NXF1-NXT1 mediates docking of the messenger ribonucleoprotein (mRNP) to, and translocation through, the NPCs by interacting with NPC proteins (termed nucleoporins or Nups), specifically with a subset of Nups enriched in phenylalanine-glycine repeats (FG-Nups) that line the NPC translocation channel (*7*–*9*). The binding of NS1 to NXF1-NXT1 prevents its docking, with its cargo mRNA, at the NPC, impairing mRNA translocation to the cytoplasm and subsequent translation (*2*–*4*). Consistent with this model, we showed that an influenza virus defective in NXF1 binding is attenuated (*3*).

A potentially analogous virulence factor from coronaviruses is the nonstructural protein 1 (Nsp1), which targets cellular processes to inhibit gene expression and down-regulate type I IFN response (*10*–*12*). Like NS1, Nsp1 is a multifunctional protein. Severe acute respiratory syndrome coronavirus (SARS-CoV) Nsp1 inhibits translation (*11*, *13*, *14*), triggers host mRNA cleavage (*13*, *15*) and decay (*10*, *11*), and induces cytokines and chemokines (*16*, *17*). While the mechanisms underlying these effects are not fully understood, specific mutants of Nsp1 in multiple coronaviruses have been shown to alter virus replication and the innate immune response. One example is the SARS-CoV Nsp1 mutant K164A and H165A, which does not promote mRNA degradation, does not inhibit translation, and induces type I IFN expression (*11*). Nsp1 from SARS-CoV-2 has also been recently shown to inhibit translation (*18*). Together, these results indicate that wild-type SARS-CoV inhibits gene expression and antiviral response and suggest that the Nsp1 protein of SARS-CoV-2 may also act at multiple levels. Here, we report a previously unknown interaction of the SARS-CoV-2 Nsp1 protein with the cellular mRNA nuclear export machinery that inhibits nuclear export of mRNA and promotes viral infection.

## RESULTS

### SARS-CoV-2 Nsp1 protein interacts with the mRNA export receptor NXF1-NXT1

We sought to determine the interacting partners of Nsp1 protein from SARS-CoV-2 (Fig. 1A) to dissect its mechanisms of action. We expressed glutathione *S*-transferase (GST)–Nsp1 in 293T cells and subjected cell lysates to immunoprecipitation followed by mass spectrometry analysis (table S1). Among the peptides identified were those derived from translation factors, which is consistent with the previously reported interactions of Nsp1 with the translation machinery (*18*, *19*). Other targets include constituents of the nuclear transport machinery such as the cellular mRNA export receptor NXF1 (Fig. 1A) and the mRNA export factor/RNA helicase UAP56 (DDX39B), which function in nuclear export of mRNAs (*20*). Early in their biogenesis, cellular mRNAs are bound by export factors such as the THO complex, and UAP56 is thought to recruit the mRNA export adaptor Aly/REF (THOC4) (*20*), which, in turn, recruits the mRNA export receptor NXF1-NXT1 (*5*, *6*). NXF1-NXT1 then mediates mRNA translocation through the NPC to the cytoplasm for translation (*5*, *6*). The Nsp1 interactions with NXF1 and UAP56 were confirmed by immunoprecipitation of Nsp1 from nuclear lysates (of cells expressing Nsp1) and Western blot analysis (Fig. 1B and fig. S1). Other members of the mRNA export pathway [THOC3 and GANP (germinal center-associated nuclear protein)] did not bind Nsp1 (Fig. 1B and table S1). We have also detected a weak binding of Nsp1 to nucleolin, whose nucleocytoplasmic distribution was previously shown to be altered by SARS-CoV Nsp1 (*21*). The binding of Nsp1 to NXF1 was confirmed in cell lysates from SARS-CoV-2–infected cells by immunoprecipitation of Nsp1 and detection of NXF1 by Western blot (Fig. 1C). Pull-down assays were then performed with purified recombinant GST-Nsp1 or GST-Nsp1 truncation mutants lacking the flexible regions flanking the β barrel domain (*22*) and endogenous NXF1-NXT1 from cell lysate. We found that Nsp1 binds to the endogenous NXF1-NXT1 and that Nsp1 (residues 13 to 129) is sufficient to mediate this interaction (fig. S2). In vitro binding assays were then carried out using purified recombinant Nsp1 and purified NXF1-NXT1 or domains of NXF1 [RNA binding domain encompassing the RNA recognition motif (RRM) and leucine-rich repeat (LRR) domains, and the nucleoporin-binding domains NTF2L and ubiquitin-associated domain (UBA)]. We determined that the interaction between Nsp1 and NXF1 is direct (Fig. 1D). No substantial binding was observed between Nsp1 and truncated mutants of NXF1 encoding the RRM-LRR or (LRR-NTF2L)-NXT1, suggesting that both domains are important for the interaction with Nsp1. Using an electrophoretic mobility assay, we also showed that the Nsp1 interaction with NXF1 does not impair the binding of NXF1 to RNA (Fig. 1E).

### Nsp1 inhibits nuclear export of cellular poly(A) RNA

**Fig. 2:**
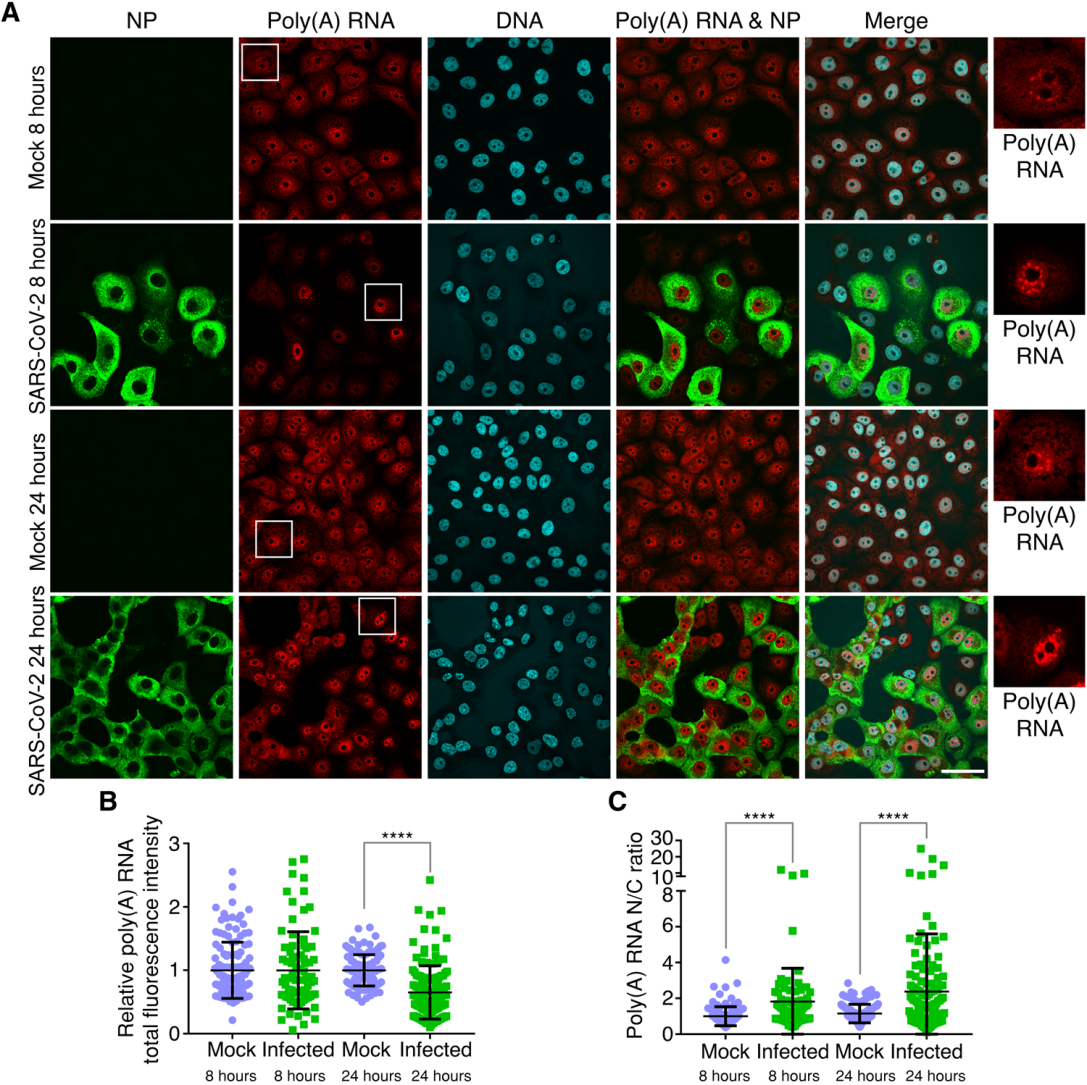
SARS-CoV-2 inhibits poly(A) RNA nuclear export. (**A**) Poly(A) RNA was assessed by RNA-FISH in Vero cells mock-infected or infected with SARS-CoV-2 at an MOI of 2 for 8 and 24 hours. Infected cells were detected with antibody against the viral NP protein. Boxed regions are enlarged and shown on the right. Scale bar, 50 μm. (**B** and **C**) Fluorescence intensities of total cellular poly(A) RNA in individual cells (B) or the N/C ratio of poly(A) RNA signals in individual cells (C) is shown for the various depicted conditions. Data represent three independent experiments. Mock, 8 hours, *n =* 126 cells; infected, 8 hours, *n =* 80 cells; mock, 24 hours, *n =* 134 cells; and infected, 24 hours, *n =* 133 cells. ****P < 0.0001.

The selection of NXF1 for follow-up studies among other hits identified by mass spectrometry was based on the simultaneous observation that SARS-CoV-2 infection inhibited nuclear export of cellular (host) mRNAs (Fig. 2) in a manner similar to that observed upon NXF1 depletion (*23*). We examined the effect of SARS-CoV-2 infection on the intracellular distribution of poly(A) RNA using fluorescence in situ hybridization (RNA-FISH) (Fig. 2). We show that cells infected with SARS-CoV-2 retain nuclear poly(A) RNA indicative of an inhibition in mRNA nuclear export, both at 8 hours and, more prominently, by 24 hours postinfection (Fig. 2A). Imaging quantification shows that total poly(A) RNA levels were not significantly altered at 8 hours postinfection, but the nuclear-to-cytoplasmic ratio (N/C ratio) of poly(A) RNA was increased (Fig. 2, B and C). Thus, SARS-CoV-2 induces nuclear export inhibition of cellular poly(A) RNA at early stages of infection. By 24 hours postinfection, total poly(A) RNA levels were reduced, and a further increase in N/C ratio of poly(A) RNA was observed (Fig. 2, B and C). These effects at 24 hours likely reflect a combination of poly(A) RNA degradation in the cytoplasm and nuclear retention of poly(A) RNA, and they correlate with high levels of Nsp1 by 24 hours postinfection (fig. S3).

**Fig. 3:**
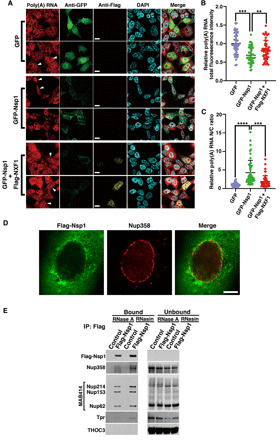
NXF1 expression reverts Nsp1-mediated mRNA export block and reduction in mRNA levels. (**A**) 293T cells were transfected with plasmids encoding GFP, GFP-Nsp1, or GFP-Nsp1 and Flag-NXF1 and then analyzed by RNA-FISH to detect poly(A) RNA and immunofluorescence to detect GFP, GFP-Nsp1, and Flag-NXF1. White arrowheads show examples for each condition. Scale bars, 10 μm. (**B**) Fluorescence intensity of total poly(A) RNA in individual cells is shown in the depicted conditions. (**C**) Fluorescence intensity of poly(A) in the nucleus and cytoplasm was determined, and the ratios of the nuclear-to-cytoplasmic signals for individual cells are shown for the indicated experimental condition. *n =* 3; ****P < 0.0001, ***P < 0.001, and **P < 0.01. (**D**) SK-N-SH cells were transfected with 3xFlag-Nsp1, and immunofluorescence microscopy was performed to detect Nsp1 and endogenous Nup358. *n =* 3. Scale bar, 5 μm. (**E**) Immunoprecipitation of Nsp1 followed by Western blot analysis shows Nsp1 interaction with certain nucleoporins, which is partially dependent on RNA.

Because Nsp1 interacts with the mRNA export receptor NXF1 (Fig. 1), we then tested whether expression of Nsp1 alone results in mRNA export block as observed during SARS-CoV-2 infection (Fig. 2). To this end, cells were transfected with plasmids encoding green fluorescent protein (GFP) or GFP-Nsp1 in the presence or absence of Flag-NXF1. While expression of GFP alone did not result in significant changes in bulk poly(A) RNA levels or intracellular distribution (Fig. 3, A to C), expression of GFP-Nsp1 caused a decrease in poly(A) RNA levels (Fig. 3, A and B) and increased N/C ratios of poly(A) RNA (Fig. 3, A and C). Both of these effects were partially rescued by expression of NXF1 (Fig. 3, A to C), indicating that Nsp1 interferes with NXF1 function by inducing nuclear export block of mRNAs, a process that concomitantly leads to decrease in mRNA levels (*24*). This phenotype of reduced levels and increased N/C ratio of poly(A) RNA induced by Nsp1 is similar to what we observed after 24 hours of SARS-CoV-2 infection (Fig. 2, A to C). However, we showed that mRNA export inhibition without altering total levels of poly(A) RNA occurred at 8 hours of infection with SARS-CoV-2 (Fig. 2, A to C), suggesting that mRNA export block precedes the reduction in mRNA levels. As a control, we expressed the Nsp9 protein of SARS-CoV-2, which has been shown to interact with nucleoporins in a proteomics study (*19*), and found no effect of Nsp9 on bulk mRNA levels or their intracellular distribution (fig. S4). Thus, these results indicate that SARS-CoV-2 inhibits host mRNA nuclear export and that the viral Nsp1 protein likely functions in this process.

### Nsp1 displaces NXF1 from the NPC, and ectopic expression of NXF1 inhibits SARS-CoV-2 infection

**Fig. 4:**
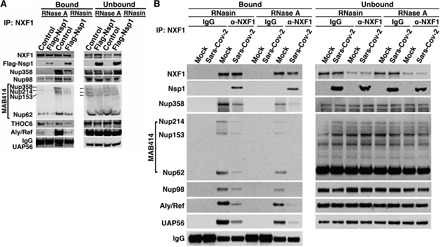
Nsp1 prevents proper interaction of NXF1 with the mRNA export machinery. (**A**) Western blot analysis of proteins immunoprecipitated with NXF1 in the presence or absence of Nsp1 or RNA reveals that the interaction of NXF1 with key mRNA export adaptors and certain Nups is inhibited by Nsp1. (**B**) 293T cells expressing the SARS-CoV-2 receptor ACE2 protein were infected with SARS-CoV-2 at an MOI of 1 for 24 hours. NXF1 was specifically immunoprecipitated from the cell lysates and followed by Western blot analysis to detect nucleoporins and mRNA export factors. Association of NXF1 with constituents of the mRNA export machinery is inhibited during SARS-CoV-2 infection.

We next examined the intracellular localization of Nsp1 from SARS-CoV-2. As shown in Fig. 3D, most of the Nsp1 is found in the cytoplasm, with a subpopulation also detected at NPCs as judged by colocalization with the nucleoporin Nup358. To further corroborate its localization at the NPC, we immunoprecipitated Nsp1 and tested whether it interacted with nucleoporins. Several Nups were detected bound to Nsp1, and this association appeared partially RNA dependent (Fig. 3E). Next, we tested the impact of Nsp1 on the association of NXF1 with several of its binding partners. Cells expressing Nsp1 were subjected to immunoprecipitation with anti-NXF1 antibody, followed by Western blot analysis. First, we tested the mRNA export adaptor Aly/REF, which recruits NXF1 to the mRNA. Nsp1 strongly decreased Aly/REF binding to NXF1, indicating competition for NXF1 interaction (Fig. 4A). In addition, the export adaptor UAP56, which recruits Aly/REF to the mRNA, does not properly interact with the NXF1-mRNA complex in the presence of Nsp1 (Fig. 4A). We also tested a member of the THO complex (THOC6) to determine whether Nsp1 affected an early step in the mRNA export pathway. We found that Nsp1 did not substantially alter THOC6 interaction with NXF1 (Fig. 4A). These findings suggest that docking of NXF1-RNA at the NPC is impaired. We show that less NXF1 binds to certain key Nups, including Nup358, Nup214, Nup153, Nup62, and, to a lesser extent, Nup98 (Fig. 4A). These results are mostly corroborated by immunoprecipitation of NXF1 during SARS-CoV-2 infection, which shows that association of NXF1 with the mRNA export machinery is also impaired (Fig. 4B). In summary, Nsp1 appears to impair the recruitment of NXF1 to the mRNA, possibly by interfering with Aly/REF function and the docking of NXF1 to the NPC.

**Fig. 5:**
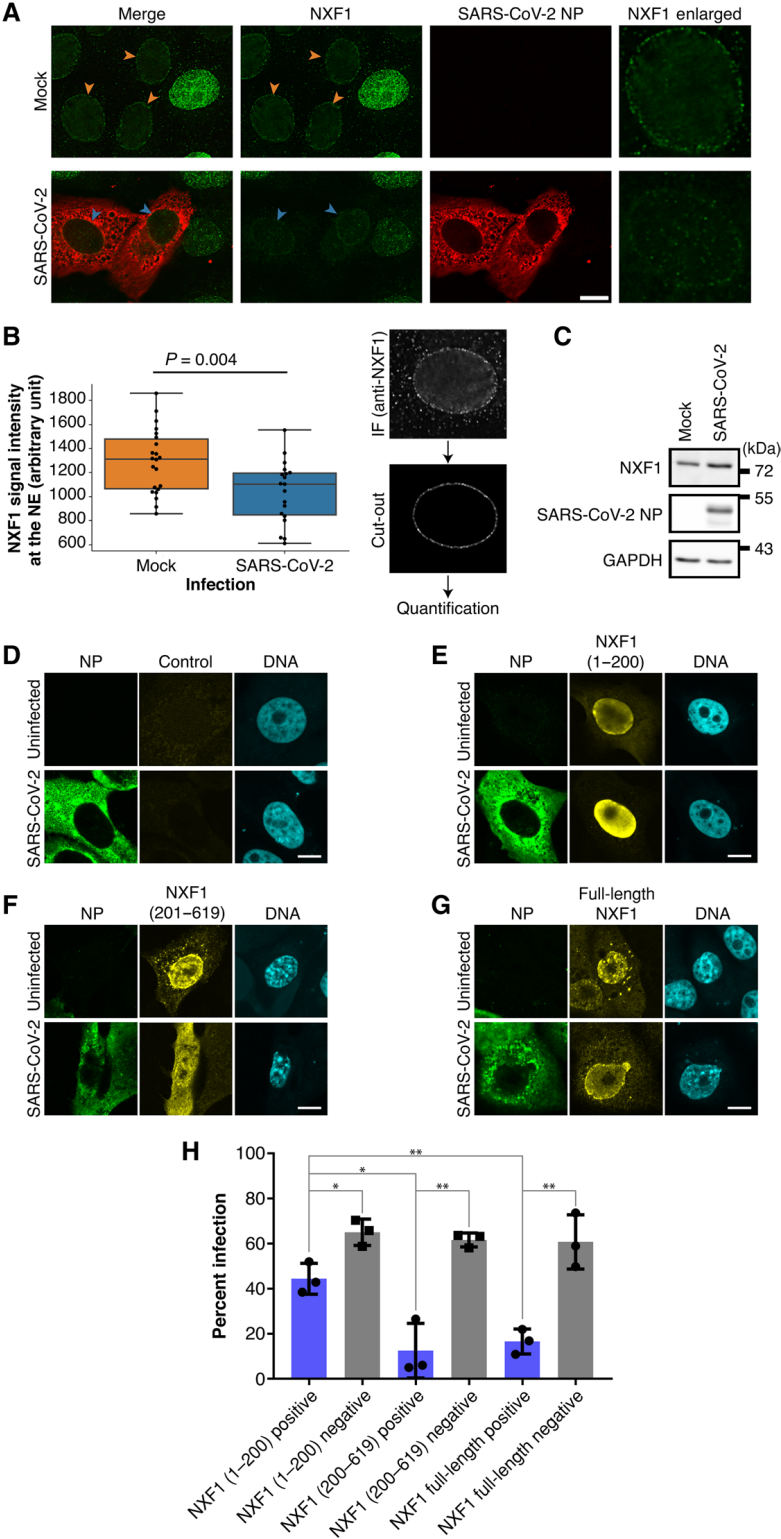
NXF1 localization at the NPC is reduced upon SARS-CoV-2 infection and increased NXF1 level prevents infection. (**A**) Vero E6 cells were infected with SARS-CoV-2 at an MOI of 1 for 48 hours. Fixed cells were treated with digitonin (1 μg/ml) to permeabilize the plasma membrane and examined by immunofluorescence microscopy to detect NXF1 and the viral NP protein. Scale bar, 10 μm. (**B**) The regions of the nuclear periphery were manually selected from equatorial sections of the nuclei in the z-stack images as indicated on the right, and the signal intensities along the NE were quantified (*n* = 22 for mock and *n* = 19 for SARS-CoV-2). Individual measurements (black dots) and the summarized box and whisker plots were overlaid (left). The difference in the NXF1 intensities at the NE between the mock and the SARS-CoV-2 infection samples was tested using Student’s *t* test. (**C**) The whole-cell extracts from Vero cells mock or SARS-CoV-2 infected were analyzed by Western blotting using anti-NXF1, SARS-CoV-2 NP, and GAPDH antibodies at 48 hours postinfection. The positions of molecular mass makers are indicated. (**D** to **G**) Vero E6 cells were transfected for 24 hours with plasmids encoding full-length Flag-tagged NXF1 or the indicated mutants of NXF1 and then infected with SARS-CoV-2 at an MOI of 1 for 24 hours. Cells were examined by immunofluorescence (IF) microscopy to detect viral NP protein and Flag-tagged NXF1. Scale bars, 10 μm. (**H**) Percentage of infected cells (NP^+^) was scored for each condition. Bars represent averages of three separate experiments, and dots represent the average of each of the three independent experiments. NXF1 (1–200), *n* = 831 cells; NXF1 (201–619), *n* = 1146 cells; and NXF1 (full-length), *n =* 1267 cells. **P < 0.01 and *P < 0.05.

Next, we infected cells with SARS-CoV-2 and assessed NXF1 localization at the NPC. To better visualize NXF1 bound to NPCs, we permeabilized cells with a low concentration of digitonin, which permeabilizes plasma membranes but leaves nuclear envelopes of most cells intact (*25*, *26*). This technique allows antibodies to access cytoplasmic proteins, including those positioned on the cytoplasmic face of the NPCs (punctate nuclear rim), but the intact nuclear envelope restricts access to nucleoplasmic proteins. In cells with an intact nuclear envelope, NXF1 is visible largely at NPCs (Fig. 5A). Using this approach, we found that NXF1 association with NPCs is significantly reduced in SARS-CoV-2–infected cells (Fig. 5, A and B). This effect is consistent with the reduced NXF1 interaction with nucleoporins seen upon Nsp1 expression (Fig. 4A) or during SARS-CoV-2 infection (Fig. 4B). This reduced levels of NXF1 at the NPC occurs without decrease in the cellular levels of NXF1 (Fig. 5C). In addition, no significant changes were observed in the localization of Aly/REF or heterogeneous nuclear RNP (hnRNP) A1 (fig. S5), suggesting that SARS-CoV-2 does not inhibit bulk nuclear transport. Thus, these findings indicate that SARS-CoV-2 infection reduces the association of NXF1 with the NPC, which impairs nuclear export of mRNAs that likely encode antiviral factors and suggest that NXF1 might have antiviral activity. Therefore, we expressed full-length NXF1 and truncated mutants expressing the N-terminal region of the RNA binding domain (NXF1 1–200) or the second half of the RNA binding module (the LRR domain) together with the NPC binding region (NXF1 201–619) in Vero E6 cells and then infected these cells with SARS-CoV-2. While NXF1 (1–200) had only a small effect on the number of infected cells, we found that less SARS-CoV-2 infection occurred in cells expressing either full-length NXF1 or the NXF1(201-619) mutant (Fig. 5, D to H).

## DISCUSSION

We have shown that infection of cells with SARS-CoV-2 inhibits host cell mRNA nuclear export. Through our analysis of this inhibitory pathway, we identified Nsp1 as a virally encoded protein capable of functioning autonomously as an mRNA export inhibitor. We propose that Nsp1 accomplishes this effect by directly binding the mRNA export factor NXF1 and reducing the interactions of NXF1 with the NPC.

Inhibiting host gene expression is a hallmark of virulence factors from many diverse viruses. Among the mechanisms used to accomplish this effect is the inhibition of host cell mRNA export. This is thought to promote infection by reducing expression of mRNAs encoding antiviral factors and increasing the availability of the translational machinery to viral mRNAs. Among the viruses that induce mRNA export inhibition is the influenza virus. For example, the virally encoded NS1 protein inhibits signaling and gene expression pathways, resulting in down-regulation of immune responses (*1*). The NS1 protein accomplishes this, in part, by binding to NXF1 and inhibiting mRNA export. We previously showed that formation of the NS1-NXF1 complex is accompanied by a reduction in the docking of the mRNPs to NPCs and translocation of the mRNA export complex to the cytoplasm (*2*–*4*).

While NXF1 is capable of binding RNA, its association with nuclear mRNAs requires adaptor proteins such as Aly/REF, suggesting that some conformational configuration of NXF1 may be required (*27*). Our results show that Nsp1 blocks the mRNA export function of NXF1 without directly interfering with its RNA binding capability, but Nsp1 disrupts NXF1 interaction with mRNA export adaptors and with the NPC. An intriguing possibility is that Nsp1 binding prevents the proper configuration of NXF1 by adaptor proteins or the formation of a productive NXF1-RNA export complex that is unable to dock and translocate through the NPC. Presumably, for Nsp1 to inhibit mRNA export, it would need to bind and alter NXF1 activity in the nucleoplasm before export. Consistent with this conclusion, while most of the detectable Nsp1 is visible in the cytoplasm, we also observe Nsp1 at, or in the vicinity of, NPCs, raising the possibility that Nsp1 may shuttle between the nucleus and cytoplasm with its steady-state distribution being predominantly cytoplasmic.

A predicted consequence of the interaction of Nsp1 with NXF1 and resulting inhibition of mRNA nuclear export is the reduced expression of host antiviral response genes and the establishment of a cellular environment that favors virus production. This idea is supported by our findings showing that ectopic expression of NXF1 significantly suppresses the effect of Nsp1 on RNA processing and nuclear export, as well as reduces SARS-CoV-2 infection. These results suggest that antagonizing Nsp1 function by interfering with its ability to bind NXF1 represents a potential means to inhibit SARS-CoV-2 replication, and thus the Nsp1-NXF1 complex is a potential target for the development of a therapeutic strategy for the treatment of COVID-19.

Our work has revealed a previously unobserved role for SARS-CoV-2 Nsp1 in altering host cell physiology during infection for the purpose of suppressing host cell gene expression. The ability of Nsp1 to bind NXF1 and block mRNA export complements its previously described role of inhibiting host cell translation through its binding to the 40*S* ribosomal subunit and obstruction of the mRNA entry tunnel (*18*). Notably, these two distinct functions of Nsp1, blocking mRNA export and translation, are performed by distinct domains within the N- and C-terminal regions of the protein, respectively. However, the outcome is conceptually similar, as in both instances, Nsp1 binding to either NXF1 or the 40*S* subunit reduces their ability to process host mRNA. These observations suggest that Nsp1 has evolved to target multiple steps in the host cell mRNA biogenesis pathway.

## MATERIALS AND METHODS

### Reagents and cell lines

Glutathione Sepharose 4B (GE Healthcare, 17-0756-01), PD-10 Desalting Column (GE Healthcare, 17-0851-01), HiTrap SP HP (GE Healthcare, 17-1151-01), Superdex 200 10/300 GL (GE Healthcare, 17-5175-01), SUPERase·In RNase (ribonuclease) Inhibitor (Thermo Fisher Scientific, AM2696), Hoechst 33258 (Thermo Fisher Scientific, H3569), ProLong Gold Antifade reagent (Thermo Fisher Scientific, P36930), and DAPI Fluoromount-G (SouthernBiotech). 293T cells and Vero E6 cells were obtained from the American Type Culture Collection (ATCC). SK-N-SH cell line was a gift from M. Hendzel (Department of Oncology, the University of Alberta). All cell lines used in this study were routinely screened for mycoplasma contamination using the Universal Mycoplasma Detection Kit (ATCC, 30-1012K).

### Antibodies

Anti-Flag M2 monoclonal antibody was used at 1:1000 dilution (Sigma-Aldrich, F1804), polyclonal rabbit anti-Nup358 (*28*) was used at 1:1000 dilution, polyclonal rabbit anti-Nup98 (*29*) was used at 1:1000 dilution, mouse NPC proteins antibody MAB414 was used at 1:1000 dilution (Abcam, ab2460), and polyclonal rabbit anti-Tpr antibodies were generated against TprC-GST (Tpr residues 2095-2348). Rabbits were immunized with the purified fusion protein at Cocalico Biologicals Inc. (Reamstown, PA). Serum containing anti-Tpr antibodies was used at 1:200 dilution, glyceraldehyde-3-phosphate dehydrogenase (GAPDH) antibody (GeneTex, GTX627408) was used at 1:10,000 dilution; monoclonal mouse anti-NXF1 antibody was used at 1:1000 dilution (Sigma-Aldrich, T1076); polyclonal rabbit anti-NXF1 antibody was used at 1:1000 dilution (Bethyl Laboratories, A303-915A); polyclonal rabbit anti-GANP antibody was used at 1:1000 dilution (Bethyl Laboratories, A303-128A); polyclonal rabbit anti-THOC3 antibody was used at 1:1000 dilution (Bethyl Laboratories, A304-870A); polyclonal rabbit anti-nucleolin antibody was used at 1:1000 dilution (Novus Biologicals, NB600-241); polyclonal rabbit anti-UAP56 antibody was used at 1:1000 dilution (Sigma-Aldrich, SAB1307254); polyclonal rabbit anti-hnRNP K antibody was used at 1:2500 dilution (GeneTex, GTX101786); monoclonal rabbit anti-tubulin antibody was used at 1:2000 dilution (Cell Signaling Technology, 2146S); anti-GFP polyclonal antibody was used at 1:250 dilution (GeneTex, GTX113617); goat anti-mouse immunoglobulin G (IgG) secondary antibody, Alexa Fluor 546 (Thermo Fisher Scientific, A-11003); anti-goat anti-rabbit IgG secondary antibody, Alexa Fluor 488 (Thermo Fisher Scientific, A-11008); anti-NXF1 monoclonal antibody was used at 1:1000 dilution (Abcam); anti-NP antibodies were used at 1:1000 dilution (GeneTex); rabbit polyclonal anti–SARS-CoV NP was used at 1:5000 dilution (produced at Mount Sinai); and rabbit anti-Nsp1 antibody was generated by Cocalico Biologicals using recombinant Nsp1 protein. Generation of Nsp1 recombinant protein and affinity purification are described in the “Protein purification” section below; Alexa Fluor 488–conjugated donkey anti-mouse IgG antibodies were used at 1:1000 dilution (Invitrogen), and Alexa Fluor 594–conjugated goat anti-rabbit IgG antibodies were used at 1:1000 dilution (Invitrogen). Horseradish peroxidase (HRP)–conjugated secondary antibodies: donkey anti-rabbit (GE Healthcare, NA934V) and sheep anti-mouse (GE Healthcare, NA931V) were used at 1:5000 dilution in most Western blot analysis except where indicated below; rabbit anti-mouse IgG (light chain specific) (D3V2A) monoclonal antibody (mAb) (HRP conjugate) (Cell Signaling Technology, 58802) was used at 1:2000 dilution; and mouse anti-rabbit IgG (heavy chain) antibody [GT881] (HRP Conjugate) (GeneTex, GTX628140-01) was used at 1:5000 dilution.

### Plasmids

Plasmids encoding NXF1(RRM-LRR-NTF2L-UBA, residues 117 to 619) and NXT1 were described previously (*3*). NXF1(LRR-NXT1, residues 203 to 549) and NXF1(RRM-LRR, residues 96 to 362) were cloned into a pGEX-4T-1 vector modified to contain a tobacco etch virus (TEV) cleavable N-terminal GST tag. Mammalian cell expression plasmid GST-tagged Nsp1 was cloned into the BamHI/XhoI sites of the pCDNA3.1+N-GST(TEV) vector. GST-tagged Nsp1, Nsp1 (1–129), and Nsp1 (13–129) were cloned into the BamHI/XhoI sites of the pGEX-6p-1 vector. Flag-tagged Nsp1 was cloned into the XhoI/SmaI sites of the pCI-neo-3XFLAG vector. GFP-tagged Nsp1 was cloned into the XhoI/BamHI sites of the pEGFP-C1 vector. Flag-tagged NXF1, NXF1 (1–200), and NXF1 (201–619) were generated as previously described (*3*). For generating antibodies, NSP1 was polymerase chain reaction amplified using pcDNA3.1-NSP1-FLAG (T.C.H. lab) as a template, and the sense 5′ CGAGCGGATCCATGGAGAGCCTTGTCCCTGG 3′ and antisense 5′-CGAGCCTCGAGCCCTCCGTTAAGCTCACGCATG-3′ oligonucleotides. NSP1 was then cloned into the pGEX6P1 (Cytiva) BamHI/XhoI sites, producing pGEX6P1-NSP1 that includes the coding sequence for a LERPHRD C-terminal extension. All expression vectors were sequenced for verification.

### Virus

Vero E6 cells were infected with SARS-CoV-2, isolate USA-WA1/2020 (BEI Resources, NR-52281), under BSL3 containment in accordance with the biosafety protocols developed by the Icahn School of Medicine at Mount Sinai. Viral stocks were grown in Vero E6 cells as described (*30*) and were verified by genome sequencing.

### Protein purification

All proteins were expressed in *Escherichia coli* Rosetta cells. NXF1(RRM-LRR-NTF2L-UBA)-NXT1 was purified as described previously (*3*). GST-NXF1(LRR-NTF2L)-NXT1, GST-NXF1(RRM-LRR), GST-NSP1, and GST-NSP1(1–129) were first purified using a Glutathione Sepharose column from cleared *E. coli* lysate. GST-NXF1(LRR-NTF2L)-NXT1 and GST-NXF1(RRM-LRR) were digested with TEV protease at 4°C overnight to remove the GST tag. NXF1(LRR-NTF2L)-NXT1 was applied to a PD10 desalting column, followed by a Glutathione Sepharose column to remove undigested protein. NXF1(RRM-LRR) was applied to a HiTrap SP column and eluted using an NaCl gradient in a buffer containing 10 mM Hepes, pH 7.0. NXF1(LRR-NTF2L)-NXT1, NXF1(RRM-LRR), GST-NSP1, and GST-NSP1(1–129) were further purified using a Superdex 200 column equilibrated with 10 mM tris (pH 8.0), 300 mM NaCl, and 0.5 mM tris(2-carboxyethyl)phosphine (TCEP). Purified proteins were concentrated and stored at −80°C.

To purify Nsp1 for generation of antibodies, Rosetta (Novagen) *E. coli* cells transformed with the pGEX6P1-NSP1 were used to inoculate 1 liter of LB media supplemented with 50 μg/ml of both ampicillin and chloramphenicol. The culture was incubated at 37°C to an OD_600_ (optical density at 600 nm) of ~0.8, followed by isopropyl-β-d-thiogalactopyranoside (IPTG) addition to 1 mM and an additional 4 hours of incubation at 37°C. Cells were then pelleted by centrifugation, and the cell pellet was stored overnight at −80°C. Forty milliliters of phosphate-buffered saline (PBS), 1% Triton X-100, and one complete EDTA-free (Roche) protease pellet were then added to the frozen cell pellet, and the mixture was vortexed until cell lysis was complete. Lysates were then sonicated to facilitate DNA shearing, and then cleared by centrifugation at 20,000*g*. To the clarified lysate, 0.5 ml of Glutathione Sepharose 4B beads (GE Healthcare) were added, and the mixture was incubated at room temperature for 2 hours with rotation. Beads were then collected by centrifugation and sequentially washed with 3 ml of various solutions including: 3× with PBS 0.1% Tween 20 (buffer A), 1× with buffer A adjusted to 500 mM NaCl, 1× with buffer A, 1× with Buffer A plus 5 mM adenosine 5’-triphosphate (ATP), 1 mM MgCl_2_, and 2× with buffer A. Each wash step included a 5-min incubation at room temperature with rotation. Buffer A (0.5 ml) plus 1 mM dithiothreitol (DTT), and 10 U of precision protease were then added to the beads to cleave Nsp1 from GST, and the mixture was incubated overnight at 4°C with rotation. Beads were then pelleted by centrifugation, and the supernatant containing Nsp1 was retained. Residual Nsp1 was then recovered by washing the beads with 0.5 ml of buffer A.

#### Purification of Nsp1 antibody

One milliliter of rabbit serum containing Nsp1 polyclonal antibodies was diluted in 20 ml of binding buffer (20 mM sodium phosphate, pH 7.0). The diluted serum was then applied to a protein A column and incubated at 4°C for 1 hour. The unbound material was removed, and the column was washed with 20 column volumes of binding buffer. Nsp1 antibody was eluted by 0.1 M glycine-HCl, pH 2.7. The elution was neutralized by 1 M tris-HCl, pH 9.0.

### GST pull-down assay

In fig. S2, GST or GST fused with full-length Nsp1, Nsp1 (1–129), or Nsp1 (13–129) were incubated with 293T total cell lysate in lysis buffer [50 mM tris, 150 mM NaCl, 1 mM EDTA, 1 mM DTT, 1% IGEPAL CA-630 (Sigma-Aldrich), 0.1 mM Na_3_VO_4_, 1 mM NaF, 1 mM phenylmethylsulfonyl fluoride (PMSF), 1× cOmplete protease inhibitor mixture (Sigma-Aldrich), and 10% glycerol (pH 7.5)] at 4°C for 4 hours. One micromolar of each protein was loaded into the pull-down assay. Beads were pelleted by centrifuging at 2300*g* for 5 min and were washed five times with 1 ml of pull-down buffer [20 mM tris, 150 mM NaCl, 1 mM DTT, and 1 mM EDTA (pH 7.5)]. Proteins remaining on the resin were extracted by sample buffer, resolved in SDS–polyacrylamide gel electrophoresis (PAGE), and then detected by Western blot. In Fig. 1D, GST or GST-NSP1 was incubated with glutathione resin in a buffer containing 20 mM Hepes (pH 7.0), 100 mM NaCl, and 0.5 mM TCEP at room temperature for 15 min. NXF1(RRM-LRR-NTF2L-UBA)-NXT1, NXF1(LRR-NTF2L)-NXT1, or NXF1(RRM-LRR) was added, and binding was allowed to proceed for 15 min at room temperature. Beads were washed extensively with the same buffer, and bound proteins were analyzed using Coomassie-stained SDS-PAGE gels.

### Cell fractionation

Cells were harvested by trypsinization and collected on ice, washed three times with cold PBS, and transferred to microfuge tubes. Cell fractionation was performed using the NE-PER Nuclear and Cytoplasmic Extraction Reagents (Thermo Fisher Scientific) according to the manufacturer’s instructions. Controls for fractionation are shown in fig. S1.

### Proteomics sample preparation

293T cells were cultured in 10-cm plates and transfected with 10 μg of GST vector control or 10 μg of GST-Nsp1 for 24 hours. Cells were then incubated in lysis buffer [50 mM tris (pH 7.5), 150 mM NaCl, 1% IGEPAL CA-630, 0.1 mM Na_3_VO_4_, 1 mM NaF, 1 mM DTT, 1 mM EDTA, 1 mM PMSF, 1× cOmplete protease inhibitor mixture, and 10% glycerol] for 30 min on ice and homogenized with vortex every 5 min. The cellular debris were removed by centrifugation. The supernatant was applied to the Glutathione Sepharose column in the presence of RNase A (10 μg/ml). After extensive washes with lysis buffer, the proteins were eluted by reduced glutathione in a buffer contain 50 mM TEAB and 4% SDS. Disulfide bonds were reduced by 20 mM DTT, followed by alkylation with 40 mM IAA. After acidification with phosphoric acid, samples were diluted with a binding buffer containing 1-to-9 volume of TEAB to methanol and loaded on S-Trap micro columns (ProtiFi). Proteins were digested with a ratio of 1:25 trypsin (Pierce) overnight at 37°C and eluted by 50% acetonitrile containing 1% formic acid. Eluted peptides were dried out in a SpeedVac and reconstituted by 2% acetonitrile in 0.1% trifluoroacetic acid (TFA).

### Liquid chromatography–tandem mass spectrometry measurement

Peptides were separated using an UltiMate 3000 RSLCnano LC system (Thermo Fisher Scientific) equipped with an in-house packed C18 column with 100-mm length and 75-μm inner diameter run at 20°C. Gradient elution was performed from 2 to 30% acetonitrile in 0.1% formic acid over 90 min. Data were acquired by a Q Exactive HF-X mass spectrometer using a method with the full mass spectrometry (MS) scans acquired at 60,000 resolution and MS/MS scans acquired at 15,000 resolution in top 15 data-dependent mode and a normalized collision energy of 28. The minimum AGC trigger was 2 × 10^5^ ions (intensity threshold 1 × 10^4^). The charge exclusion was applied to exclude unassigned and charge 1, 7, 8, and >8 species, and dynamic exclusion was applied for 30 s. Data were searched using human reference database from UniProt using Proteome Discoverer 2.4. Peptides with a minimum of six amino acid residues with trypsin/P specificity were identified. Protein N-terminal acetylation and carbamidomethyl of cysteine were used as fixed modifications, and methionine oxidation was used as a variable modification.

### Immunoprecipitation and Western blot

In Figs. 1B and 3E, 293T cells were cultured in 10-cm plates and transfected with 10 μg of 3XFLAG vector control or 10 μg of 3XFLAG-Nsp1 using jetOPTIMUS (Polypuls, 117-07). In Fig. 1B, cells were then fractionated as described above. The nuclear fraction (Fig. 1B) or total cell pellet (Fig. 3E) was lysed in 50 mM tris (pH 7.5), 150 mM NaCl, 1% IGEPAL CA-630, 0.1 mM Na_3_VO_4_, 1 mM NaF, 1 mM DTT, 1 mM EDTA, 1 mM PMSF, 1× cOmplete protease inhibitor mixture, and 10% glycerol for 30 min on ice and homogenized with vortex every 5 min. Lysates were centrifuged at 13,000*g* for 10 min to remove cellular debris. The supernatant was applied to anti-FLAG M2 magnetic beads (Sigma-Aldrich) in the presence of RNasin (1 U/μl) or RNase A (10 μg/ml) for binding overnight at 4°C. Beads were washed five times with lysis buffer at 4°C. Then, proteins were eluted using 3×Flag peptide (APExBIO A6001). The eluted fractions were mixed with 2× sample buffer [125 mM tris (pH 6.8), 10% glycerol, 2% SDS, and 0.0025% bromophenol blue] and subjected to 10% SDS-PAGE, followed by Western blot. For Figs. 1C and 4B, 293T cells expressing the SARS-CoV-2 receptor ACE2 protein were infected with SARS-CoV-2 at a multiplicity of infection (MOI) of 1 for 24 hours. Cell lysates and immunoprecipitation were performed as in Fig. 1B above except that 2 μg of Nsp1 or NXF1 antibodies were used. Protein A was then used to precipitate anit-Nsp1 antibody, and protein G was used to precipitate anti-NXF1 antibody. Western blot analysis was performed to detect the indicated proteins. The secondary antibody used in Fig. 1C to detect Nsp1 was mouse anti-rabbit IgG (heavy chain) antibody [GT881] (HRP conjugate) (GeneTex, GTX628140-01) at 1:5000 dilution, as the molecular weight of Nsp1 is close to the light chain. For the Western blot performed in Fig. 4B, rabbit anti-mouse IgG (light chain specific) (D3V2A) mAb (HRP conjugate) (Cell Signaling Technology, 58802) was used at 1:2000 dilution for probing anti-NXF1 antibody and Nups recognized by MAB414. The remaining antibodies were detected with HRP-conjugated secondary antibodies: donkey anti-rabbit (GE Healthcare, NA934V) and sheep anti-mouse (GE Healthcare, NA931V) at 1:5000 dilution.

For NXF1 immunoprecipitation in Fig. 4A, 293T cells were grown in 10-cm plates and transfected with 10 μg of 3×Flag vector control or 10 μg of 3×Flag-Nsp1 for 24 hours. The nuclear lysate was then centrifuged at 13,000*g* for 10 min to remove cellular debris. The supernatant was applied to protein G beads bound to either rabbit IgG or rabbit NXF1 antibody and incubated overnight at 4°C. After five washes with lysis buffer, the proteins were eluted with 2× sample buffer and subjected to 8% SDS-PAGE.

### RNA-FISH and fluorescence microscopy

Poly(A) RNA-FISH, immunofluorescence microscopy, and image quantification were performed as we previously described (*31*, *32*). Images depicting infected cells with SARS-CoV-2 in Fig. 2 were analyzed in a confocal laser scanning microscope, Zeiss LSM 880 Meta (Carl Zeiss Microimaging), fitted with a Plan Apochromatic 40×/1.4 oil objective. Images were collected at 16 bits and with a resolution of 1024 × 1024 pixels. For the experiment performed in Fig. 4C, Vero E6 cells were seeded into 24-well plates and transfected with 200 ng of the expression vectors indicated in the figure using TransIT-LT1 (Mirus Bio) according to the manufacturer’s instructions. On the following day, cells were either mock infected or infected with SARS-CoV-2 at an MOI of 1 in viral growth media. At 24 hours postinfection, cells were fixed and processed for poly(A) RNA-FISH and immunofluorescence as we previously described (*31*, *32*). Anti-Flag monoclonal antibody and rabbit polyclonal anti-SARS-CoV NP were used in these experiments.

For the experiment performed in Fig. 3D, SK-N-SH cells were seeded in a 24-well plate containing 12-mm-diameter coverslips. Cells were transfected with the Flag-NSP1 plasmid and Lipofectamine 3000 (Invitrogen) for 24 hours, fixed with 3.7% formaldehyde in PBS for 30 min at room temperature, and stored in PBS at 4°C. Cells were permeabilized with 0.1% Triton X-100 in PBS for 10 min at room temperature and washed three times with PBS-T (PBS + 0.1% Tween 20), followed by blocking with PBS-T + 1% bovine serum albumin (BSA) for 20 min at room temperature. Cells were incubated with a primary antibody solution containing a mouse anti-FLAG M2 monoclonal antibody (1:1000 dilution) and rabbit anti-Nup358 antibodies (1:1000 dilution) in PBS-T + 1% BSA for 1 hour at room temperature, and washed three times with PBS-T + 0.1% BSA for 5 min each at room temperature. The samples were further incubated with a secondary antibody solution containing Alexa Fluor 488–conjugated donkey anti-mouse IgG antibodies (1:1000 dilution; Invitrogen) and Alexa Fluor 555–conjugated donkey anti-rabbit IgG antibodies (1:1000 dilution; Invitrogen) in PBS-T + 1% BSA for 1 hour at room temperature and washed three times with PBS-T for 5 min each at room temperature. The coverslips were mounted on DAPI Fluoromount-G (SouthernBiotech).

For data shown in Fig. 3 (A to C), 293T cells were seeded in a 24-well plate containing 12-mm-diameter coverslips. Each well was transfected with 0.5 μg of GFP or 0.5 μg of GFP-Nsp1 and/or cotransfected with 0.5 μg of 3xFlag-NXF1 for 24 hours. Cells were then fixed with 4% formaldehyde in PBS for 15 min at room temperature, followed by permeabilization with PBST (PBS + 0.5% Triton X-100) for 5 min at room temperature. After blocking with PBST + 5% BSA overnight at 4°C, cells were incubated with anti-Flag M2 monoclonal antibody at 1:200 dilution and anti-GFP polyclonal antibody at 1:250 dilution in PBS [containing 0.2% Triton X-100, 1 mM DTT, and RNAsin (200 U/ml)] for 1 hour at room temperature and washed once with PBS containing 0.2% Triton X-100 and three times with PBS. Cells were then subjected to poly(A) RNA-FISH as described above. Next, cells were labeled with secondary antibodies—goat anti-mouse IgG Alexa Fluor 546 and goat anti-rabbit IgG Alexa Fluor 488 for 1 hour at room temperature—and washed three times with PBS, stained with Hoechst 33258 (1 μg/ml) for 10 min, and briefly washed with PBS. Coverslips were mounted in ProLong Gold Antifade reagent.

For the experiment performed in Fig. 5A, Vero cells were seeded in a 24-well plate containing 12-mm-diameter coverslips. Cells were infected with SARS-CoV-2 (MOI = 1) for 48 hours, fixed with 3.7% formaldehyde in PBS for 10 min at room temperature, and stored in PBS at 4°C. The localization of the NXF1 and the NP proteins was probed in a sequential manner because different membrane permeabilization conditions for each primary antibody were applied for immunostaining. In the first round of staining, cells were permeabilized with digitonin (1 μg/ml) (Calbiochem) in PBS for 10 min on ice and washed three times with PBS-T, followed by blocking with PBS-T + 1% BSA for 20 min at room temperature. The samples were incubated with a mouse anti-NXF1 monoclonal antibody (1:1000 dilution; Abcam) in PBS-T + 1% BSA for 1 hour at room temperature and washed three times with PBS-T + 0.1% BSA for 5 min each at room temperature. The samples were further incubated with Alexa Fluor 488–conjugated donkey anti-mouse IgG antibodies (1:1000 dilution; Invitrogen) in PBS-T + 1% BSA for 1 hour at room temperature, washed three times with PBS-T for 5 min each at room temperature, and fixed with 3.7% formaldehyde in PBS for 10 min at room temperature. For the second round of staining, cells were then permeabilized with 0.1% Triton X-100 in PBS for 10 min at room temperature, blocked, and subjected to the immunodetection using rabbit anti-NP antibodies (1:1000 dilution; GeneTex) as a primary antibody and Alexa Fluor 594–conjugated goat anti-rabbit IgG antibodies (1:1000 dilution; Invitrogen) as a secondary antibody. The coverslips were mounted on DAPI Fluoromount-G (SouthernBiotech). Cells were imaged using DeltaVision Elite (GE Healthcare) equipped with the PlanApo N 60×/1.42 numerical aperture oil objective (Olympus) as a series of z-stacks at intervals of 0.24 μm. Images were deconvolved with the deconvolution module of the SoftWoRx software (GE Healthcare) under a “conservative” setting. Deconvolved images were further processed (crop, normalization) with ImageJ (National Institutes of Health).

For the NXF1 quantification at the NE (Fig. 4B), a single equatorial section from a nucleus (*n* = 22 for mock and *n* = 19 for SARS-CoV-2) from z-stack images was selected, and the area of the NE was isolated by manually placing boundaries within two pixels of the cytoplasmic and nucleoplasmic sides of the NE, creating a ring-shaped region containing the NE-associated NXF1-specific signal. The signal intensity of NXF1 at the NE was calculated as the sum of the NXF1 signal in the region of the nuclear periphery normalized to the area of the ring region. The difference in the NXF1 intensities at the NE between the mock and the SARS-CoV-2 infection samples was tested using Student’s *t* test.

### Electrophoretic mobility shift assay

Poly(U) 15-mer RNA (100 nM) labeled with Alexa Fluor 488 at the 5′ end was mixed with NXF1(RRM-LRR) and GST-NSP1(1–129) in a buffer containing 10 mM tris (pH 8.0), 100 mM NaCl, 0.5 mM TCEP, 8% glycerol, and SUPERase·In RNase Inhibitor (0.5 U/μl). The mixtures were incubated at room temperature for 10 min. Samples were separated on a 5% native PAGE gel. RNA was visualized with the Typhoon FLA 9000 biomolecular imager (GE Healthcare).

## SUPPLEMENTARY MATERIALS

Supplementary material for this article is available at http://advances.sciencemag.org/cgi/content/full/7/6/eabe7386/DC1
